# Hydroxyurea-associated digital gangrene: a case report and narrative review of reported cases and emerging pathophysiology

**DOI:** 10.1186/s12959-026-00887-0

**Published:** 2026-06-19

**Authors:** Angela Misic, Ghadi Ghanem, Saeed Sadeghi, Matthew Carroll

**Affiliations:** 1 Department of Medicine, David Geffen School of Medicine, University of California, Los Angeles, 757 Westwood Plaza, Los Angeles, CA 90095 USA; 2 Department of Hematology-Oncology, David Geffen School of Medicine, University of California, Los Angeles, 757 Westwood Plaza, Los Angeles, CA 90095 USA

**Keywords:** Polycythemia vera, Myeloproliferative neoplasm, Hydroxyurea, Microvascular thrombosis, Digital gangrene, Digital ischemia, Drug-induced vasculopathy

## Abstract

**Background:**

Hydroxyurea (HU) is a first-line oral cytoreductive agent for selected patients with myeloproliferative neoplasms (MPNs), including polycythemia vera (PV), due to its efficacy and tolerability. Although cutaneous ulceration is a recognized complication of long-term HU exposure, HU-associated digital gangrene is rare, and upper-extremity involvement in PV has not previously been reported.

**Case presentation:**

We describe the first case of HU-associated digital gangrene in a patient with PV. A 72-year-old man with well-controlled Janus kinase 2 (JAK2)–positive PV, treated with HU for more than a decade, developed painless dry gangrene of the right index and middle fingers. Vascular imaging showed no significant arterial occlusion or embolic source. Hematologic parameters remained within target ranges, and the workup for autoimmune disease, infection, and hypercoagulability was unremarkable. HU was discontinued, and the ischemia stabilized without surgical intervention. With no alternative etiology identified, delayed HU-associated vasculopathy was suspected.

**Conclusions:**

Our literature review identified three previously reported cases of HU-associated digital gangrene, though limited to the lower extremities – two in chronic myeloid leukemia (CML) and one in sickle cell disease (SCD). In each case, gangrene developed after prolonged HU exposure, alternative etiologies were not substantiated, and stabilization or clinical improvement followed HU withdrawal. The present case aligns with this pattern while extending the reported phenotype to well-controlled PV and upper-extremity digits. Given the small number of reported cases, the pathophysiology remains incompletely defined and is largely extrapolated from studies of more frequently described HU-associated ulceration, histopathologic reports of HU-related tissue injury, and in vitro studies of HU effects on endothelial and circulating cells. Plausible mechanisms include cumulative endothelial injury, localized thrombo-occlusive microvascular dysfunction, impaired vascular and cutaneous repair, and interaction with PV-related microvascular susceptibility. Clinicians should include HU-associated vasculopathy in the differential diagnosis of otherwise unexplained digital ischemia, as prompt drug cessation may limit progression and improve digit salvage.

## Introduction

Hydroxyurea (HU) is a cornerstone cytoreductive therapy in the treatment of myeloproliferative neoplasms (MPNs), including polycythemia vera (PV), owing to its established efficacy, oral administration, and generally favorable tolerability profile [1, 2]. With prolonged exposure, however, HU has been associated with a spectrum of mucocutaneous toxicity, most notably chronic lower-extremity ulceration, which has been reported in up to 9% of patients receiving long-term therapy and is often refractory to standard wound care [3–6]. Severe acral ischemic complications appear to be far less common, although they have been described. Among these, digital ischemia and gangrene are of particular clinical significance because of their potential to culminate in irreversible necrosis, functional compromise, and amputation [7–10].

Here, we report what is, to our knowledge, the first case of HU-associated digital gangrene in a patient with well-controlled Janus kinase 2 (JAK2)–positive PV and upper-extremity involvement. We also review and synthesize the limited published literature to contextualize this presentation within the emerging spectrum of HU-associated vasculopathy, examine plausible pathophysiologic mechanisms, and highlight practical considerations for diagnosis and management.

## Case presentation

A 72-year-old man with well-controlled JAK2-positive PV presented with progressive discoloration of the right-hand digits. His medical history was notable for non-obstructive coronary artery disease and cutaneous malignancies, including melanoma. For more than a decade, his PV had been managed with aspirin 81 mg daily, hydroxyurea 500 mg twice daily, and intermittent phlebotomy, with overall good hematologic control, minimal symptom burden, and no documented concern for nonadherence.

**Fig. 1:**
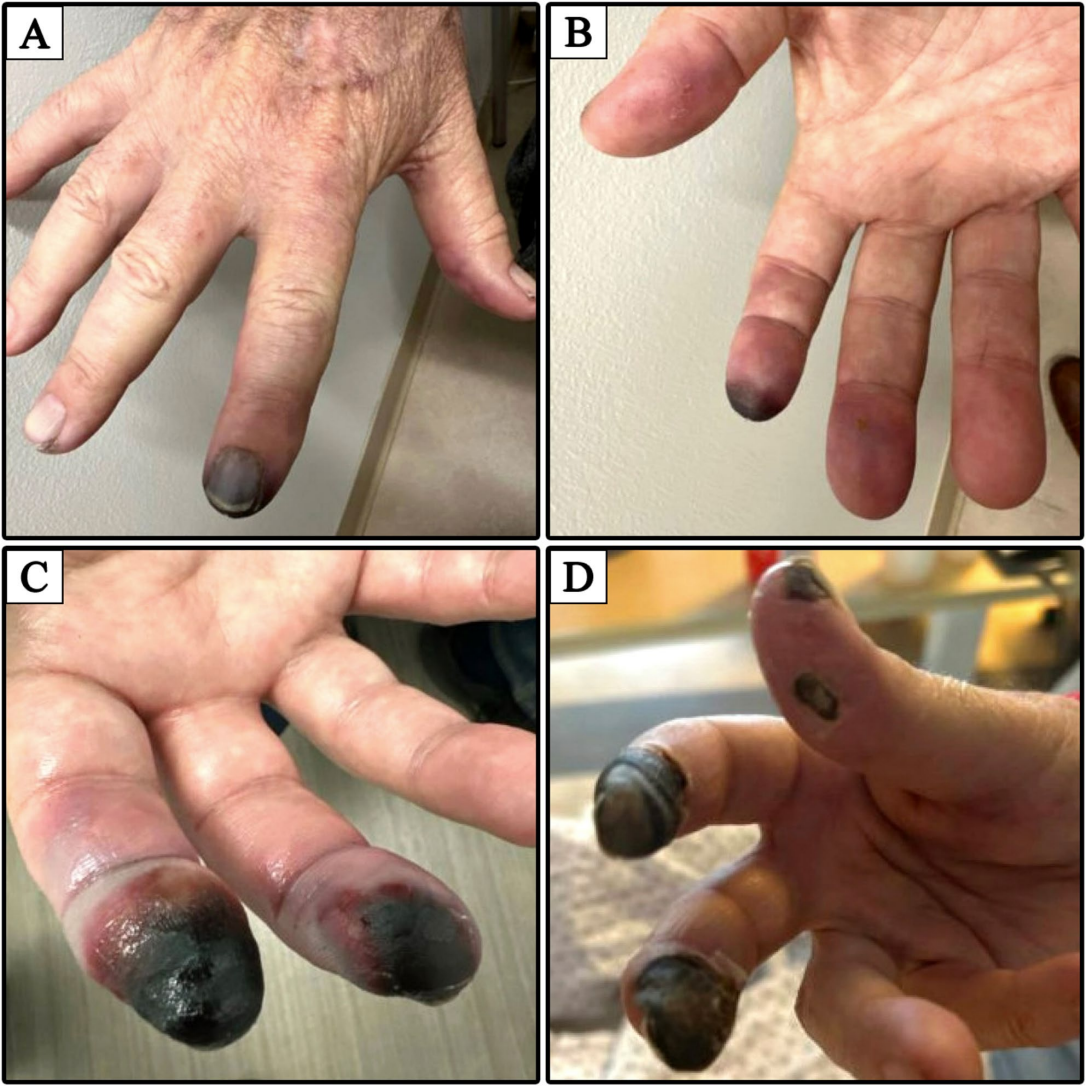
Progressive necrosis of the right hand digits in a patient with HU-associated digital gangrene. (**A–D**) Serial clinical photographs demonstrate progression from early distal dusky discoloration of the right index finger with preserved proximal perfusion (**A–B**) to ischemic changes involving additional fingertips and the development of dry gangrene (**C–D**); panels are arranged in chronological order from earliest (**A**) to latest (**D**)

Approximately two months prior, he noted intermittent redness and numbness of the right index finger. He had no known prior history of Raynaud phenomenon, vasospastic episodes, or other acral ischemic symptoms before the current presentation. Over the following month, the fingertip became progressively dusky and darkened without associated pain or systemic symptoms. By the time of emergency department presentation, he had developed dry gangrene of the right index finger extending just beyond the distal interphalangeal (DIP) joint, gangrenous change along the radial aspect of the right middle finger distal to the DIP joint, and evolving ischemic change at the tip of the right thumb (Fig. 1). On exam, the affected digits were cold, with capillary refill greater than five seconds, but radial and ulnar pulses remained palpable. There was no erythema, fluctuance, drainage, or other evidence of superimposed infection.

Initial laboratory studies showed mild leukocytosis with a white blood cell (WBC) count of 13.25 × 10⁹/L, hemoglobin of 13.5 g/dL, hematocrit of 42.3%, and a platelet count of 322 × 10⁹/L. Inflammatory markers were within normal limits, including a C-reactive protein (CRP) of 2 mg/L and erythrocyte sedimentation rate (ESR) of 5 mm/hr.

Vascular evaluation revealed no hemodynamically significant large-vessel stenosis or occlusion on upper-extremity arterial duplex ultrasonography. Computed tomography angiography of the chest and transthoracic echocardiography with bubble study showed no embolic or other proximal vascular source. Magnetic resonance imaging of the right hand demonstrated soft-tissue and marrow changes consistent with digital necrosis.

Further evaluation for alternative etiologies was unrevealing. Autoimmune and hypercoagulability testing, including antinuclear antibody, antineutrophil cytoplasmic antibodies, antiphospholipid antibodies, complement levels, and cryoglobulins, were negative or within normal limits. A low-titer cold agglutinin was not felt to be clinically significant. Laboratory markers of platelet activation were not obtained. Blood cultures were negative. Bone marrow biopsy confirmed persistent myeloproliferative neoplasm without leukemic transformation.

Throughout the diagnostic workup, multiple subspecialty teams were engaged, including hematology, rheumatology, infectious diseases, orthopedic hand surgery, and vascular surgery. Each concurred that no unifying alternative etiology could be identified, and in light of this, the gangrenous changes were attributed to a rare but recognized delayed complication of long-term hydroxyurea therapy. HU was subsequently discontinued during hospitalization. Given his thrombosis risk and the need for continued cytoreduction, transition to ruxolitinib was planned.

Given the absence of infection or unstable tissue on exam, and after evaluation by the orthopedic hand surgery team, the patient elected to pursue conservative management with autoamputation. Subsequent follow-ups revealed no progression of the necrosis since hospitalization and HU discontinuation.

## Case-based literature review

To better characterize our case amidst existing literature, we conducted a narrative review on HU-associated digital gangrene and proposed mechanisms. A search of PubMed and Google Scholar for English-language, peer-reviewed publications using combinations of relevant terms, including “hydroxyurea,” “digital ischemia,” “gangrene,” “cutaneous toxicity,” and “myeloproliferative neoplasms,” identified three published cases of HU-associated digital gangrene, two in chronic myeloid leukemia (CML) and one in sickle cell disease (SCD), summarized in Table 1 [7, 9, 10]. Despite arising in different hematologic disorders, these reports share several recurring features.

Across the three cases, gangrene developed only after prolonged HU exposure, with treatment durations ranging from approximately two to four years before symptom onset. In all cases, the underlying hematologic disease was well controlled at the time of ischemic presentation, and conventional vascular risk factors, such as diabetes, smoking, or significant large-vessel atherosclerotic disease, were notably absent or not substantiated. The earliest manifestations were also broadly similar, beginning with localized ischemic skin changes such as erythema, blistering, or discoloration, affecting the toes or nail folds, before progressing insidiously over weeks to months to dry gangrene.

Another recurring feature was the absence of a more convincing alternative explanation. Large-vessel occlusion was not demonstrated in the published cases, and competing diagnoses, including vasculitis, peripheral angiopathy, or other thrombotic and inflammatory drivers, were variably excluded across reports [7, 9, 10]. Taken together, these findings suggest that the gangrenous process arose in the setting of otherwise unexplained distal ischemia rather than as a direct manifestation of uncontrolled underlying disease.

Most importantly, all reported cases demonstrated stabilization or clinical improvement after HU discontinuation, despite differences in adjunctive management. Cessation of HU was associated with gradual clinical improvement over a period of months, with the earliest improvement observed by approximately two months and stabilization or resolution documented by nine months [7, 9, 10]. This recurring pattern of delayed onset during long-term exposure, unrevealing evaluation for alternative causes, and arrest of progression after drug withdrawal provides the strongest comparative support for a clinically meaningful association between HU and digital gangrene.

Our case aligns closely with this pattern while extending it in two important ways. First, it occurred in a patient with well-controlled JAK2-positive PV. Second, it involved the upper extremity digits rather than the toes. The patient developed insidious digital gangrene after more than a decade of continuous HU therapy, with hematocrit, platelet count, and leukocyte counts within therapeutic targets and no prior history of thrombosis. Vascular imaging excluded large-vessel obstruction and embolic sources, and autoimmune, infectious, and hypercoagulability evaluations were unrevealing. Notably, ischemic progression halted after HU discontinuation, mirroring the trajectory observed in the previously published cases.

These features broaden the recognized clinical spectrum of HU-associated vasculopathy and strengthen the rationale for considering HU in the differential diagnosis of otherwise unexplained digital ischemia. This is particularly significant as we believe this complication is underrecognized, given that digital ischemia can be mistakenly attributed to disease progression or other vascular pathologies. For instance, in all reported cases, HU had been continued for significant periods of time despite worsening digital cutaneous changes and necrosis until its association was retrospectively appreciated and drug withdrawal led to stabilization or improvement [7, 9, 10].

**Table 1:** Published reports of hydroxyurea-associated digital gangrene

Study (year)	Age, sex	Indication + HU exposure	Co-therapy	Site	Key negative workup	Management	Outcome
Yasuda et al. (2000)	53 F	CML; HU 3 yrs (1.5 g/day)	IFN-α	Toes (bilat), heels, L malleolus	No large-vessel occlusion; coagulation factors normal; biopsy negative for vasculitis	Transmetatarsal amputation; HU stopped	Resolved over ~ 3 months
Leo et al. (2002)	49 M	CML; HU 4 yrs	IFN-α (prior; stopped 1 year before)	All ten toes	No peripheral angiopathy on duplex sonography; biopsy negative for vasculitis; cryoglobulins negative; no diabetes; X-ray negative for osteomyelitis	1 toe amputated; HU stopped	No progression at 9 months
Bhangu et al. (2024)	35 F	SCD; HU 2 yrs (up-titrated to 2 g/day)	None	All ten toes	ABI/CT inconclusive; vasculitis serologies non-diagnostic (tests NR); biopsy showing fibrinous debris	HU stopped; red cell exchange; HBOT	Improved by ~ 2 months; resolved by 9 months
Misic et al. (2026)	72 M	PV (JAK2+), HU 12 yrs (500 mg BID)	Intermittent phlebotomy	R index and middle fingers; evolving R thumb tip	No large-vessel occlusion on duplex/CTA; echo negative for embolic source; autoimmune/APS panel negative; cryoglobulins negative; cultures negative	HU stopped; planned switch to ruxolitinib; conservative management (autoamputation)	Progression halted after HU cessation

## Discussion

Although HU-associated cutaneous toxicity is well recognized, digital gangrene appears to be a rare but likely underrecognized adverse effect, in part because underreporting and diagnostic uncertainty may obscure its identification [9, 11, 12]. For instance, even HU-associated ulceration, a more commonly described cutaneous adverse effect, has been associated with delayed recognition, with a reported median diagnostic delay of eight months [11]. This has been attributed largely to substantial clinical overlap with the patients’ underlying disease states, such as sickle cell disease, as well as other vascular and inflammatory conditions, including venous insufficiency, peripheral arterial disease, and vasculitis. Based on the available literature and our experience, a similar diagnostic challenge is present in cases of digital gangrene. Although documented infrequently compared with HU-associated ulceration, these events can have catastrophic consequences of irreversible tissue necrosis and limb loss [7, 9, 11, 12].

The pathophysiology of HU-associated digital gangrene remains poorly defined. Because so few cases have been reported, current mechanistic understanding is necessarily extrapolated from related evidence, including clinical reports of HU-associated ulceration, histopathologic descriptions of HU-related tissue injury, and in vitro studies of HU effects on different cell types [7, 9, 11–13]. Any proposed mechanism must therefore be interpreted cautiously. At present, the most plausible explanation is a multifactorial process involving cumulative endothelial toxicity, small-vessel occlusive or thrombotic change, and impaired tissue repair, rather than a single discrete pathogenic pathway.

One mechanism particularly relevant to the present case is progressive endothelial injury associated with prolonged HU exposure. HU exerts its therapeutic effect through noncompetitive inhibition of ribonucleotide reductase, suppressing deoxyribonucleic acid (DNA) synthesis and arresting DNA replication in rapidly proliferating hematopoietic cells [1, 11, 14]. However, this antiproliferative effect is not entirely restricted to the hematopoietic cells [1, 11]. Experimental models have demonstrated that endothelial cells are similarly susceptible to HU exposure in a time- and dose-dependent manner. At concentrations approximating therapeutic plasma levels, endothelial cells remain viable yet demonstrate significant functional impairment, including progressive loss of adhesion and structural integrity. This effect is more pronounced at greater concentrations of HU and longer times of exposure [1, 13, 14]. As such, this effect appears to be predominantly cytostatic rather than apoptotic, characterized by impaired proliferative capacity and reduced endothelial renewal rather than immediate cell death [1, 13]. This pattern of dose- and time-dependent endothelial susceptibility is of clinical interest because reported cases of HU-associated digital gangrene generally occur after years of treatment rather than shortly after initiation [7, 9, 10]. Importantly, while the HU plasma concentration thresholds associated with endothelial functional impairment described in experimental models already overlap with plasma concentrations achievable during standard therapeutic dosing, this exposure may be further amplified in older patients or those with impaired renal clearance, as HU is substantially renally eliminated [1, 11]. Although this pharmacokinetic overlap does not establish direct clinical toxicity, it supports the plausibility of cumulative endothelial stress during prolonged treatment [11, 13]. It is also important to note that while these endothelial effects have not been directly validated in clinical cases of HU-associated gangrene, the clinical pattern of delayed, refractory HU-associated ulceration, often with necrotic features, as well as gangrene, provides clinical support for this mechanistic model.

A second mechanism that may bridge HU exposure to digital gangrene is localized thrombo-occlusive microvascular dysfunction [8, 12]. HU is therapeutically used to reduce thrombotic risk through cytoreduction; however, its effects on circulating cells and the endothelium may also create conditions favorable to focal microvascular obstruction [11]. For example, its inhibition of DNA synthesis has been demonstrated to induce megaloblastic erythropoiesis and macrocytosis [3, 11]. In one experimental model, HU-associated erythrocyte enlargement was associated with an approximately 39% increase in cell volume and a 12% increase in minimum diameter, with reduced deformability and increased resistance during passage through 3-µm filter pores used as an in vitro capillary model [11, 15]. These findings suggest that HU-induced macrocytosis may impair erythrocyte transit through narrow distal microvascular beds, promoting local stasis and tissue hypoxia [3, 11].

Beyond rheologic effects, HU may also alter adhesive interactions within the microcirculation. HU exposure has been associated with increased expression and phosphorylation of Lutheran/basal cell adhesion molecule (Lu/BCAM) on erythrocytes, resulting in increased adhesion to laminin within the vascular wall. Importantly, this effect has been reported independently of JAK2V617F allele burden, supporting a drug-related effect rather than one attributable solely to the underlying myeloproliferative disease. HU has also been associated with increased expression of CD147, another adhesion-related erythrocyte membrane protein [16]. At the endothelial level, HU-induced upregulation of intercellular adhesion molecule-1 (ICAM-1) may further promote leukocyte adhesion and vascular trapping [14].

Together, these changes create a plausible sequence in which macrocytosis impairs flow, erythrocyte and leukocyte adhesion amplify microvascular stasis, and focal thrombo-occlusion develops in distal tissues with limited collateral perfusion. This model is supported by histopathologic reports of HU-associated necrotic or ulcerative lesions demonstrating fibrinoid thrombi, small-vessel occlusion, and thrombo-occlusive vasculopathy [8, 12]. This mechanism may also help explain the distal distribution of HU-associated tissue injury, with gangrene preferentially affecting the tips of toes and fingers, and ulceration occurring most often in similarly vulnerable acral sites and around the malleoli, where microvascular flow, mechanical stress, and limited collateral perfusion converge [3, 11].

A third mechanism is failure of cutaneous and vascular repair, which may explain why HU-associated tissue injury often progresses until the drug is discontinued [3, 11]. This repair failure is a direct extension of HU’s primary pharmacologic action. By inhibiting ribonucleotide reductase, HU depletes deoxyribonucleotide pools, arrests cells in S phase, and limits DNA synthesis in proliferating cells required for tissue maintenance [1, 11]. In the skin, this impairs basal keratinocyte renewal and dermal fibroblast activity, reducing epidermal regeneration, collagen production, and dermal tensile strength [11, 17]. In the vasculature, HU similarly impairs endothelial proliferation, migration, and angiogenesis [13, 14]. Experimental studies have shown reduced endothelial proliferative and invasive capacity, inhibition of capillary-like structure formation, and suppression of pro-angiogenic pathways, including VEGF, bFGF, Ang1, and HIF-1α signaling [11, 13]. Thus, once ischemic or cutaneous injury develops, HU may prevent the coordinated epithelial, stromal, and vascular repair response required for healing. Clinically, this model is supported by the refractory nature of HU-associated ulcers and necrotic lesions, which, in the vast majority of cases, fail to improve with local wound care, antibiotics, dressings, compression, or debridement while HU is continued [3, 8, 11]. In contrast, healing is reported after HU withdrawal in the majority of cases, suggesting that ongoing exposure acts as a sustained pharmacologic barrier to repair [3, 9, 11].

Because the present case arose in a patient with PV, another mechanistic consideration is whether HU may have interacted with disease-specific microvascular susceptibility [18, 19]. Moreover, an important alternative explanation is that the ischemic process may have been driven by the underlying PV itself rather than by HU exposure [7, 20]. PV is intrinsically associated with endothelial dysfunction, abnormal blood cell adhesion, and thrombotic predisposition, and these vascular abnormalities may persist even when hematocrit and platelet counts are adequately controlled [18, 19]. Qualitative abnormalities in erythrocyte, platelet, and leukocyte behavior—including enhanced endothelial adhesion and impaired microcirculatory flow—may continue to confer microvascular risk independent of overt cytosis [7, 16, 19]. As discussed, experimental and translational studies suggest that while erythrocytes in PV exhibit increased endothelial adhesiveness, HU may further modulate these adhesion-related pathways, including Lu/BCAM- and CD147-mediated interactions [16, 21]. These observations raise the possibility that HU may not act as an isolated insult, but rather amplify an already pro-adhesive and microvascularly vulnerable state, which is plausible.

At the same time, several features of the present case make PV alone a less compelling explanation. To the best of our knowledge, the reported cases of PV-associated digital ischemia and gangrene have occurred in the setting of marked erythrocytosis, extreme thrombocytosis, or other evidence of uncontrolled disease, where hyperviscosity and overt thrombotic occlusion provide a more direct mechanistic basis for tissue loss [7, 19]. In contrast, our patient’s hematologic parameters remained within therapeutic target range at symptom onset and throughout progression of the gangrene, arguing against active disease-driven hyperviscosity or cytosis-mediated vascular occlusion as the primary driver [3, 9]. Moreover, the delayed onset after prolonged HU exposure, together with stabilization after drug withdrawal, favors a drug-associated contribution [7, 9, 11]. Thus, PV likely provided a baseline microvascular vulnerability, but the temporal and clinical features in this case support HU as, at least, a precipitating or amplifying factor.

These uncertainties underscore an important limitation of the current literature. Because HU-associated digital gangrene is rare, mechanistic conclusions are drawn from isolated case reports and from extrapolation out of adjacent HU toxicity literature, particularly chronic ulceration, and in vitro studies. Direct histopathologic, vascular, and translational data specific to digital gangrene remain extremely limited. Further study is therefore needed to clarify whether the dominant process is endothelial toxicity, microthrombosis, impaired wound healing, or a combination of these mechanisms, and whether patients with myeloproliferative neoplasms carry disease-specific susceptibility that would alter our current approach to pharmacotherapy.

From a practical standpoint, this case, on the background of existing literature, emphasizes the need to consider HU-associated vasculopathy in patients receiving long-term therapy who develop otherwise unexplained acral ischemia. Early manifestations may be subtle, including numbness and discoloration, and may precede overt gangrene by weeks to months. The diagnostic evaluation should exclude more common competing causes, particularly disease progression, peripheral arterial disease, embolic ischemia, systemic vasculitis and coagulopathies when clinically suspected. In practice, this may require vascular imaging, assessment for proximal embolic source, and directed serologic evaluation [22–26].

When HU-associated gangrene is suspected, and alternative explanations are not substantiated, withdrawal of HU is the key management step based on the currently available evidence. Across the limited published literature, as in the present case, cessation of HU has been associated with stabilization or improvement of ischemic lesions [7, 9, 10]. For patients who require ongoing cytoreductive therapy, subsequent treatment should be individualized on the basis of the underlying hematologic disorder, thrombotic risk, comorbidities, and tolerance of available alternatives.

## Conclusion

This case broadens the reported phenotype of HU-associated digital gangrene by showing that it may arise in the setting of well-controlled JAK2-positive PV and involve the upper extremities. Taken together with the limited existing literature, it suggests that HU-associated vasculopathy should remain in the differential diagnosis when patients receiving long-term therapy develop otherwise unexplained acral ischemia, particularly because early recognition and drug withdrawal may help limit further progression. At the same time, the rarity of this complication and the uncertainty surrounding its pathogenesis underscore the need for further study to clarify underlying mechanisms, define susceptibility factors, and better inform diagnosis and management.

## Data Availability

No datasets were generated or analysed during the current study.

## Abbreviations

HU: Hydroxyurea
MPN: Myeloproliferative neoplasm
PV: Polycythemia vera
ET: Essential thrombocythemia
CML: Chronic myeloid leukemia
SCD: Sickle cell disease
FDA: Food and Drug Administration
WBC: White blood cell
CRP: C-reactive protein
ESR: Erythrocyte sedimentation rate
ANA: Antinuclear antibody
ANCA: Antineutrophil cytoplasmic antibodies
APS: Antiphospholipid syndrome
CTA: Computed tomography angiography
DIP: Distal interphalangeal
MRI: Magnetic resonance imaging
LDH: Lactate dehydrogenase
RBC: Red blood cell
MCV: Mean corpuscular volume
NO: Nitric oxide
BCAM: Basal cell adhesion molecule
PAD: Peripheral arterial disease
